# Composite Active Packaging Film of Zein, Citric Acid, and FBTE: Fabrication, Property Analysis and Its Preservation Impact on Lard

**DOI:** 10.3390/foods15132317

**Published:** 2026-06-30

**Authors:** Yi Yuan, Xinrui Luo, Jiaxin Wei, Li Yang, Cuntang Wang

**Affiliations:** 1 College of Food and Bioengineering, Qiqihar University, Qiqihar 161006, China; 2 Plant Food Processing Technology Research Center, Ministry of Education, Qiqihar 161006, China

**Keywords:** zein, Fu brick tea ethanol extract, active packaging, lipid oxidation inhibition, biodegradable film

## Abstract

Advancing bio-based intelligent packaging is crucial for improving food protection and prolonging storage duration. This research fabricated zein/citric acid (Z/CA) hybrid films embedded with Fu brick tea ethanol extract (FBTE) via a casting technique. The films were comprehensively assessed for their physicochemical attributes, mechanical strength, antioxidant and antimicrobial efficacy, and biodegradability. Findings indicated that FBTE inclusion notably improved film hydrophobicity and antioxidative power, elevating DPPH and ABTS radical quenching efficiencies by 83.75% and 89.33%, respectively. However, the incorporation of high-dose FBTE (8 wt%) increased the water vapor permeability by 57.19%, which was unfavorable for moisture barrier performance, and SEM showed surface morphology and phase segregation at 8 wt% FBTE. These hybrid films demonstrated specific antibacterial behavior against Gram-positive microorganisms. Importantly, the 6 wt% FBTE film exhibited obvious inhibitory effects on lipid oxidation, decreasing peroxide value by 68%. Observations during the 28-day soil burial degradation test revealed gradual morphological changes in the film samples. These results illustrate that Z/CA/FBTE hybrid films have possess potential application value in food active packaging.

## 1. Introduction

In the food industry, the storage stability and shelf life of foodstuffs are directly governed by the intrinsic properties of packaging materials. Conventional petroleum-derived plastic packaging materials have been extensively utilized owing to their lightweight nature, low cost and superior processability [1]. Nevertheless, such materials are non-biodegradable and tend to trigger persistent plastic pollution. Meanwhile, residual additives within packaging matrices may migrate into food matrices, thereby posing severe threats to food safety and human health [2]. Accordingly, bio-based active packaging materials integrated with biodegradability, antioxidant capacity and antibacterial activity have emerged as a core research hotspot in the field of lipid preservation packaging [3]. Natural polymers are promising substrates for fabricating eco-friendly packaging films. Among them, zein stands out for its outstanding film-forming capacity, tunable barrier performance and high biosafety, which renders it widely applicable to the development of food-active films [4,5]. Previous studies have validated that the incorporation of polyphenols and natural tea extracts into zein matrices can effectively ameliorate the drawbacks of neat zein films, including poor mechanical toughness and weak antibacterial efficacy, and endow resultant composite films with prominent lipid antioxidant capacity [5,6].

Nevertheless, prominent research gaps remain regarding zein/Fu brick tea extract composite films. Most current investigations merely assess the antioxidant and preservation efficacy of films toward liquid lipids, whereas few studies deliberately develop active packaging systems tailored for solid animal lipid preservation. Solid lard is susceptible to oxidative rancidity triggered by oxygen, microorganisms and metal ions during storage. Its solid state imposes distinctive requirements on packaging films, including satisfactory flexibility, favorable interfacial adhesion and the sustained controlled release of antioxidant substances, which cannot be fully satisfied by existing film formulations. To address the aforementioned bottlenecks, the present work fabricates a novel multifunctional composite film dedicated to solid lard preservation, aiming to achieve long-term eco-friendly preservation of solid lipids.

As a major byproduct generated during corn starch processing, zein is abundant in hydrophobic amino acids. Continuous films with stable microstructure and favorable light transmittance can be formed via intermolecular hydrogen bonds and hydrophobic interactions among zein chains [6,7]. However, neat zein films suffer from inherent deficiencies, such as inferior flexibility, low elongation at break and weak humidity resistance. Their mechanical and barrier properties deteriorate drastically under high-humidity environments, limiting their practical applications in food storage packaging [8]. To tackle these limitations, multiple modification strategies, including physical blending, chemical crosslinking and small-molecule plasticization, have been adopted to optimize film performance [9]. Citric acid is recognized as a green and efficient crosslinking modifier; it rearranges the molecular chain arrangement of zein through intermolecular crosslinking and plasticization, improving film flexibility and structural compactness while simultaneously enhancing mechanical strength and antibacterial activity. Therefore, citric acid serves as a preferable modifier for zein-based films [10]. Although the fundamental performance of citric acid-modified zein films has been optimized, single-modified systems exhibit insufficient antioxidant capacity and fail to retard long-term lipid oxidative rancidity. Hence, natural bioactive components are required to construct multifunctional composite matrices.

Fu brick tea is a typical microbially fermented tea. Its extracts are rich in tea polyphenols, tea polysaccharides and functional small molecules, which confer robust and stable antioxidant and antibacterial activities with excellent biosafety, making Fu brick tea extracts high-quality natural functional additives for food-active packaging [11,12,13,14]. Numerous previous studies have incorporated green tea and black tea extracts into polymeric film systems, verifying that tea extracts can efficiently scavenge free radicals, suppress microbial proliferation and delay food oxidative deterioration [15,16]. However, two critical limitations exist in current tea extract-incorporated composite films. First, most investigations employ unfermented or lightly fermented tea extracts, whose bioactive components are unstable and prone to oxidative deactivation, resulting in short-term preservation effects. Second, existing studies on Fu brick tea extract composite films only conduct basic physicochemical characterizations, without targeted preservation research against the unique oxidation characteristics of solid lipids, and thus cannot resolve the rancidity issue of solid lipids during storage. Compared with ordinary tea extracts, microbially fermented Fu brick tea extracts contain more stable polyphenol constituents and deliver prolonged antioxidant activity, which meets the demand of long-term food preservation. Even so, technologies for stable immobilization and controlled release of Fu brick tea polyphenols within zein films still require further optimization.

At present, the modification of bio-based films using natural extracts derived from agricultural wastes has become a mainstream research direction for sustainable packaging. Extracts isolated from grape pomace, fruit and vegetable residues and various teas have been proven to boost the bioactivity of packaging films [17,18,19,20]. However, conventional zein–polyphenol and zein–tea extract composite systems commonly suffer from rapid degradation of active ingredients, uncontrollable release kinetics and poor film stability, which lead to inconsistent preservation performance and short service life, making them unsuitable for the long-term storage of solid lipids. Distinct from conventional single-blending modification strategies, this study innovatively introduces citric acid to construct zein composite matrices. Relying on the synergistic stabilization effect of the matrix, bioactive polyphenols from Fu brick tea are immobilized, overcoming the technical barrier of facile oxidation and rapid deactivation of natural polyphenols and realizing sustained and controllable release of active substances [21]. This design differs from traditional lipid encapsulation films and single tea extract-modified films, as it not only alleviates the poor stability of pure polyphenol films but also fills the research void of existing active lipid packaging materials incapable of adapting to solid lipid systems.

Solid animal lipids (e.g., lard) readily undergo oxidative rancidity induced by oxygen, microorganisms and metal ions during storage and transportation, accompanied by elevated peroxide values, off-flavor formation and quality deterioration, posing a great challenge for solid lipid preservation [22]. Existing domestic and international research on active lipid packaging predominantly focuses on liquid lipid preservation, with film formulations designed according to the fluidity and high oxidizability of liquid lipids. Such studies completely ignore the unique deterioration rules of solid lipids, including solid interfacial characteristics, low fluidity and slow continuous oxidation. Furthermore, current zein-based active films and polyphenol composite films lack targeted optimization for the specific oxidation mechanisms of solid lipids, and no specialized packaging systems have been developed for solid lipid preservation. To fill these research gaps, the present work constructs a ternary composite film system composed of Fu brick tea extract, zein and citric acid. The mechanisms underlying the effects of varying Fu brick tea extract dosages on film mechanical properties, barrier performance, antioxidant capacity and antibacterial activity are systematically explored. The structure–activity relationship between film microstructures and solid lipid preservation capacity is elucidated, with special emphasis on verifying the inhibitory effect of composite films against the elevation of peroxide value in solid lard throughout storage. This research breaks through the limitations of poor stability of conventional polyphenol films and narrow adaptability of lipid packaging materials, and provides novel theoretical foundations and technical support for the exploitation of dedicated biodegradable active packaging materials for solid lipids.

## 2. Materials and Methods

### 2.1. Materials

The experimental components and their origins were as follows: Jingwei Fu brick tea was sourced from Jingwei Fu Brick Tea Co., Ltd. (Xi’an, China); lard was bought from Jiefangmen Market (Qiqihar, China); zein, yeast extract, DPPH, and ABTS were provided by Shanghai Aladdin Biochemical Technology Co., Ltd., Guangdong Huankai Microbial Technology Co., Ltd. (Shanghai, China), and Sigma-Aldrich Chemical Company (St. Louis, MO, USA), respectively; all other chemical substances were obtained from Tianjin Kaitong Chemical Reagent Co., Ltd. (Tianjin, China).

### 2.2. Preparation of FBTE

The isolation of polyphenols from Fu brick tea was carried out according to a previously published technique [23] with adjustments. The resulting FBTE was kept at −20 °C prior to its subsequent utilization.

### 2.3. Preparation of Z/CA/FBTE Composite Film

Z/CA/FBTE films were synthesized using the solution casting technique following the protocol of Wang et al. [23]. A certain mass of zein was precisely weighed. The zein was dissolved with a volume fraction of 80% ethanol solution. The zein suspension at a concentration of 8 g/100 mL was prepared. The suspension was magnetically stirred for 15 min until the zein was completely dissolved 0.1 g/g glycerol (*w/w*: glycerol mass/zein-based mass) and 0.2 g/g citrate acid (*w/w*: citrate mass/zein-based mass) were added to the zein suspension. The mixture was magnetically stirred for 15 min, and subsequently the mixture was heated in an 80 °C water bath for 30 min. Subsequently, the Z/CA film-forming fluid was obtained. FBTE with different mass fractions (2 wt%, 4 wt%, 6 wt%, 8 wt%, based on dry zein mass) was added to the Z/CA film-forming solution. The Z/CA/FBTE film forming solution was then stirred at 80 °C for 20 min. The membrane-forming fluid was subsequently placed in an 80 °C water bath for 30 min. A 120 mm diameter polyethylene ring was fixed to a glass plate covered with detached paper. Then, 20 g of composite membrane liquid was poured into the polyethylene ring and dried at 55 °C for 12 h. The dried film-forming solution was uncovered. The film was placed in a dryer with a temperature of 25 °C and a relative humidity of 56.8% (NaBr of saturated solution). The prepared film was left for 72 h to determine the indicators of the film.

These films were designated as Z/CA/FBTE-0%, Z/CA/FBTE-2%, Z/CA/FBTE-4%, Z/CA/FBTE-6%, and Z/CA/FBTE-8% based on the specific quantity of FBTE incorporated.

### 2.4. Property Characterization of Z/CA/FBTE Composite Film

#### 2.4.1. Fourier-Transform Infrared (FTIR) Spectroscopy

The Fourier-transform infrared spectroscopy of Z/CA/FBTE films was evaluated following the procedure established by Qin et al. [24]. The film samples were dried after the equilibration treatment and then placed on an attenuated total reflection (ATR) accessory for analysis. The test conditions were set as a temperature of 25 °C, a wavenumber range of 4000–650 cm^−1^, and a resolution of 4 cm^−1^, and the number of scans was 32.

#### 2.4.2. X-Ray Diffraction (XRD) Analysis

The apparatus settings were defined at 45 kV and 200 mA. The tilt was configured so that 2θ varied between 5° and 60°. The measurement velocity was adjusted to 4° per minute [24].

#### 2.4.3. Scanning Electron Microscopy (SEM)

The fine-scale morphology of the Z/CA/FBTE coatings was examined via SEM (S-4300, Hitachi, Tokyo, Japan) following the protocol outlined by Wang et al. [25].

#### 2.4.4. Thickness Measurement

The dimensions of Z/CA/FBTE hybrid layers were evaluated via a micrometer [26].

#### 2.4.5. Optical Properties

Following the approach described by Gao et al. [27], the L*, a*, and b* chromatic parameters for film specimens were evaluated. The optical density metric was measured based on the protocol established by Sukhija et al. [28].

#### 2.4.6. Determination of Mechanical Properties

The mechanical properties of Z/CA/FBTE hybrid films were evaluated following the methodology described by Huang et al. [29].

#### 2.4.7. Determination of Water Vapor Transmittance (WVP)

The WVP of Z/CA/FBTE integrated films was evaluated according to the methodology established by Gao et al. [27].

#### 2.4.8. Water Contact Angle (WCA) Measurement

The contact angle of the film specimens was measured using the procedure established by Lei et al. [30].

#### 2.4.9. Total Phenolic Content and Radical Scavenging Properties

Based on the protocol developed by Xie et al. [31], the overall phenolic concentration and radical inhibitory potential of the Z/CA/FBTE samples were analyzed. The total phenolic quantity was determined and represented as gallic acid equivalents (mg GAE/g dry weight). Antioxidant efficacy was measured via ABTS and DPPH tests, with the antioxidant strength presented as percentage scavenging efficiencies.

#### 2.4.10. Determination of Antibacterial Activity

The antimicrobial activity of the composite films was performed using the method described by Meng et al. [32] with some modifications.

#### 2.4.11. Soil Degradability

The soil biodegradability of the composite film was assessed following the procedure of Su et al. [33].

#### 2.4.12. Peroxide Value (POV) Determination

POV are identified following the approach proposed by Gao et al. [27]. Every specimen is measured three times, and the results are averaged.

#### 2.4.13. Data Analysis

Research findings were presented as averages ± standard deviations. Statistical distinctions between categories were evaluated via Duncan’s multiple range procedure utilizing SPSS 26.0 (SPSS Inc., Chicago, IL, USA). Every graph was generated using Origin 2022 (OriginLab Corporation, Northampton, MA, USA).

## 3. Results and Discussion

### 3.1. FTIR Analysis of the Thin Film

**Figure 1:**
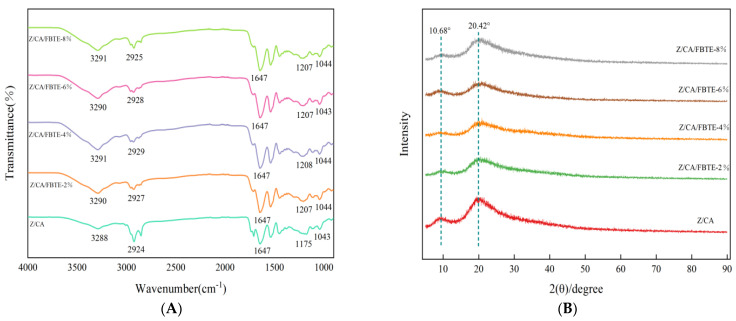
FTIR (**A**) plus XRD profiles (**B**) for Z/CA/FBTE-0%, Z/CA/FBTE-2%, Z/CA/FBTE-4%, Z/CA/FBTE-6% and Z/CA/FBTE-8% specimens.

FTIR techniques were employed to determine functional group compositions and examine possible chemical interactions in these films. As illustrated in Figure 1A, the FTIR spectrum of the Z/CA/FBTE film exhibited no novel absorption peaks relative to the Z/CA film. The lack of new peaks suggests that integrating FBTE did not cause the creation of additional chemical bonds, indicating a physical blending within the composite [34]. The Z/CA film demonstrated a broad peak at 3288 cm^−1^ and a sharp peak at 2924 cm^−1^, corresponding to N–H and O–H stretching vibrations, respectively [35,36]; the 1647 cm^−1^ peak was associated with C=O stretching (amide I) [37]; peaks at 1175 cm^−1^ and 1045 cm^−1^ indicated C–O stretching [38]. The peak at 1207 cm^−1^ corresponds to the C–O–C ether bond stretching of polyphenols in FBTE, while the peak at 1043 cm^−1^ is attributed to the C–O stretching of polysaccharide hydroxyl groups. The broadening of these peaks with increasing FBTE dosage verifies the formation of intermolecular hydrogen bonds between polyphenols and zein. FTIR patterns resembling those of the Z/CA film were identified in all four Z/CA/FBTE films with incorporated extract. However, compared to the Z/CA film, blending FBTE into the zein polymer resulted in weaker and broader peaks at higher frequencies. This could be due to the interaction between FBTE and zein molecules weakening the intermolecular bonds of the zein polymer [39]. Analogous research indicates that in investigating functional chitosan/zein films with Rosa roxburghii Tratt leaf extract from natural deep-eutectic solvents, as extract concentration increased, these peaks became broader and sharper [40].

### 3.2. XRD Analysis of the Film

X-ray diffraction (XRD) techniques were utilized to evaluate the structural characteristics of the prepared films [41]. The XRD patterns of these layers are displayed in Figure 1B. The Z/CA film exhibited clear peaks at 2θ values of 10.68° and 20.42°. Nevertheless, as FBTE was integrated, the amplitudes of these diffraction peaks at the particular 2θ positions gradually diminished, resulting in a subsequent reduction in the film’s crystalline nature. This occurrence occurred since FBTE interfered with the secondary organization of zein and competed with citric acid for cross-linking positions, thus lowering the crystallinity of the hybrid film [42]. No additional diffraction peaks appeared following the inclusion of FBTE, indicating excellent compatibility between zein, citric acid, and FBTE [43]. This finding corresponds to the results of Kumar et al. [41]. When the water chestnut peel extract was blended into the chitosan framework at different concentrations, no novel peaks were observed in the XRD profiles of the specimens.

### 3.3. Analysis of the Microstructure

**Figure 2:**
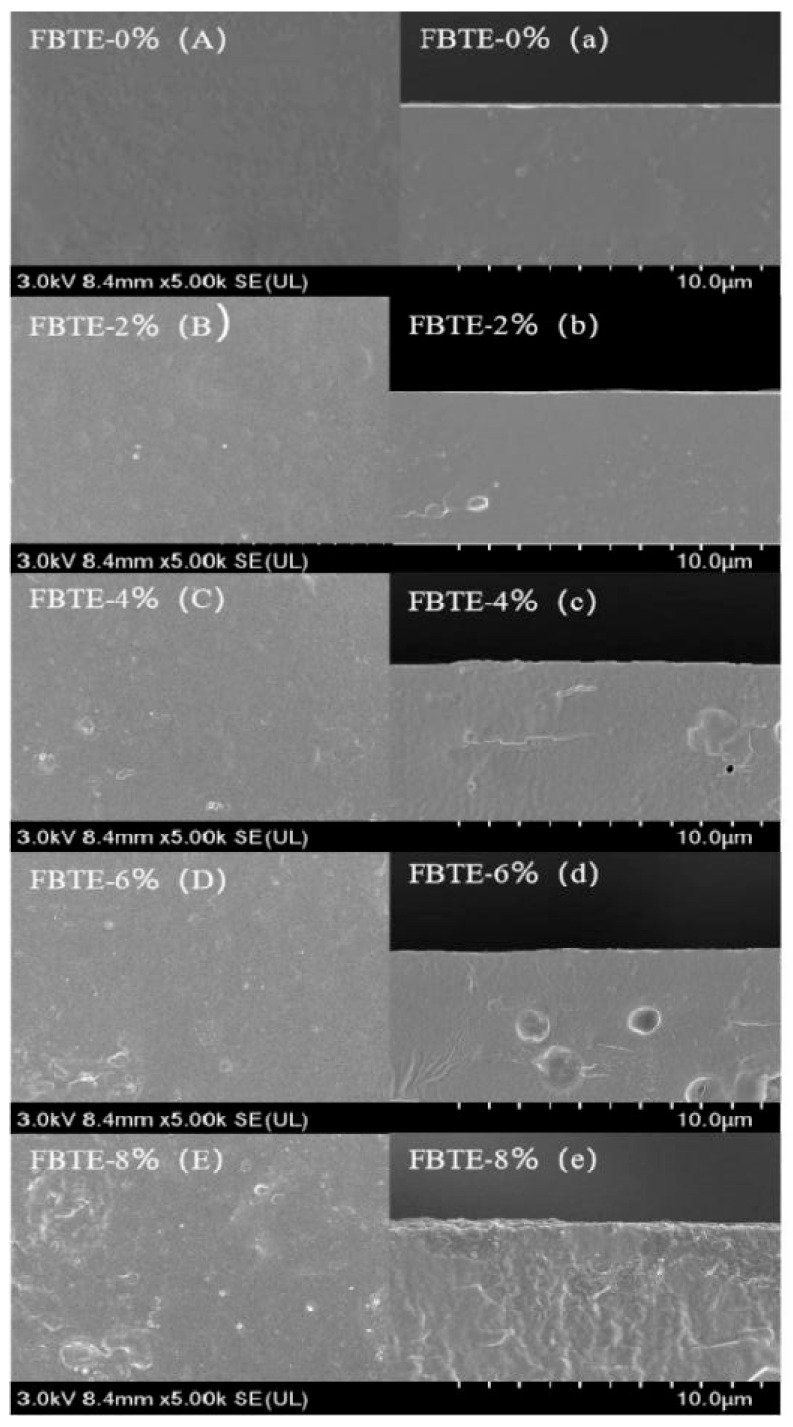
SEM photographs displaying the surface (**A**–**E**) and cross-section (**a**–**e**) of the film.

SEM allows for the direct evaluation of the surface and internal architecture of zein–polyphenol complexes via secondary electron imaging [44]. Figure 2 illustrates SEM surface and cross-sectional views of the Z/CA/FBTE multi-layer films across various FBTE concentrations (0–8%). As illustrated in Figure 2A–E,a–e, the surface and cross-sectional morphology of these films remain fairly smooth, lacking distinct particulate or porous features, maintaining a homogeneous appearance. When the FBTE ratio increases from 2 wt% to 8 wt%, the film’s surface and cross-sectional framework become distinctly more porous, marked by a growing quantity and scale of voids, alongside the emergence of insoluble agglomerates. This indicates that at high FBTE loadings, polyphenol agglomeration and poor interfacial compatibility between components induce the generation of agglomerated particles and internal pores within the films. Excessive FBTE breaks down the continuous zein protein network, serving as the direct cause of elevated porosity [45]. In a concurrent study regarding the development and characterization of active smart packaging films composed of cassava starch and Lycium barbarum anthocyanins (LRAs), it was noted that films with reduced LRA levels possessed a dense structure, whereas an excessive amount of LRAs impaired the starch orientation and produced a rougher surface quality [46].

### 3.4. The Thickness, Color, and Opacity of the Composite Film

Film thickness has a substantial effect on its optical transmittance, water vapor transmission rate (WVP), and mechanical properties, as documented by You et al. [47]. Table 1 illustrates the effect of different FBTE concentrations on the thickness, color, and translucency of Z/CA/FBTE composite films. As the FBTE ratio increases from 0 wt% to 8 wt%, the film thickness undergoes a major 28.08% growth (*p* < 0.05). This observation highlights the crucial function of FBTE quantity in defining the thickness of Z/CA/FBTE films. Similarly, prior studies have demonstrated that in films synthesized from corn starch and polyvinyl alcohol, the inclusion of purple sweet potato and red cabbage extract anthocyanins causes a matching rise in thickness with increasing extract proportions [48].

Optical characteristics, encompassing opacity and colorimetry, hold significant value within food packaging contexts [25]. Within the colorimetric framework, L* defines brightness, a* signifies the red–green dimension, and b* represents the yellow–blue axis. With the Z/CA/FBTE-0% composite film as the control, the total color difference (ΔE*) of all modified films increased significantly with rising FBTE content. The ΔE* value of the 2% group was 3.68, representing a color difference visible to the naked eye, whereas the 8% group showed an extremely remarkable color discrepancy with a ΔE* of 21.07. This tendency is highly consistent with the combined changing patterns of L*, a* and b*. Meanwhile, the opacity of the films rose continuously from 4.59% to 8.22% as FBTE loading increased. This phenomenon is mainly attributed to enhanced light scattering caused by the polyphenols, pigments and fine particles contained in FBTE [49]. Although higher opacity can effectively suppress the UV-induced deterioration of photosensitive products, the simultaneous drop in light transmittance and obvious color deviation may reduce the aesthetic appearance of packaged products, thereby exerting a negative influence on consumer preference.

### 3.5. Analysis of Mechanical Properties

**Figure 3:**
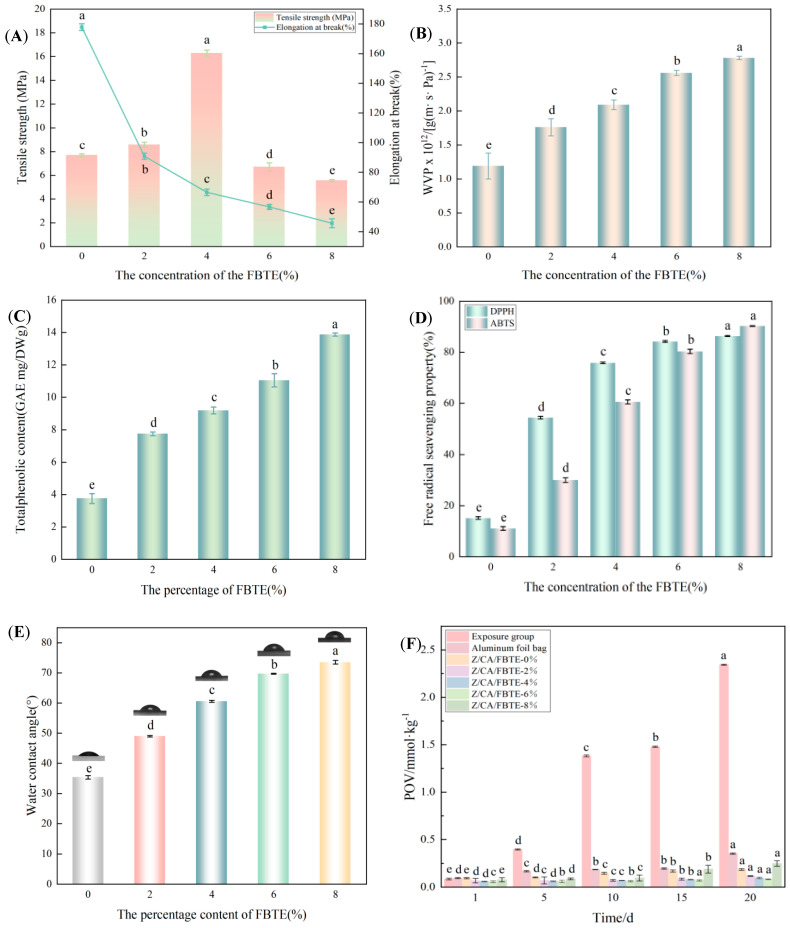
The mechanical characteristics (**A**), WVP (**B**), TPC (**C**), antioxidant potency (**D**), WCA (**E**) and POV (**F**) of the Z/CA/FBTE-0%, Z/CA/FBTE-2%, Z/CA/FBTE-4%, Z/CA/FBTE-6% and Z/CA/FBTE-8% films. ^a–e^ Values are given as means + SD. Different letters in the figure indicate significant difference (*p* < 0.05).

Tensile strength (TS) and elongation at break (EAB) are crucial indicators utilized to characterize the tensile capacity and ductility of the film [50]. Figure 3A illustrates the variations in the TS and EAB for the Z/CA/FBTE composite films across various FBTE concentrations. As the extract volume fraction rises, the TS initially climbs before dropping, whereas the EAB exhibits a steadily declining pattern. As the FBTE concentration escalates from 0 wt% to 4 wt%, the TS slowly improves. Nevertheless, when the proportion subsequently grows to 8 wt%, relative to the base film, the TS drops by 27.74%. Concerning the EAB relative to the Z/CA/FBTE-0% film, the Z/CA/FBTE-8% film demonstrates a reduction of 74.64%. These findings suggest that a higher level of the extract causes a reduction in mechanical rigidity. This occurrence might be attributed to intense cross-linking between proteins and citric acid induced by a high density of polyphenols, which subsequently diminishes the regularity of the polymer network framework [51]. Comparable investigations have demonstrated that when rose polyphenol extract (RPE) is blended into the biodegradable active packaging film synthesized from sodium alginate and gelatin, the mechanical attributes of the film diminish following the inclusion of RPE [52].

### 3.6. Water Vapor Permeability (WVP)

Barrier performance represents a steady-state metric for degradable packaging substrates, while WVP serves as a specific assessment for evaluating moisture barrier attributes of packaging films [53]. Figure 3B demonstrates the influence of FBTE concentration on the WVP data of composite films. As FBTE levels rise, the WVP measurement of the material steadily increases. When extract concentration reaches 8 wt%, the WVP value surges by 57.19%. This phenomenon likely arises from the disruption of protein frameworks caused by polyphenol incorporation, which potentially creates additional micro-pore pathways, thereby enhancing water vapor diffusion and increasing the overall WVP [54]. Notably, the increase in WVP caused by FBTE incorporation is a major limitation of these composite films for practical applications. A higher water vapor permeability accelerates moisture exchange between the internal packaged environment and the external surroundings, thereby shortening the shelf life of lipid-rich foods and weakening the preservation performance of the active packaging. Analogous studies have observed that in the development of polyvinyl alcohol-based antioxidant nanocomposite films via impregnation with nano-kaolin and pomegranate peel polyphenols, incorporating pomegranate peel extract notably elevates the WVP value of the resulting film [55].

### 3.7. Antioxidant Activity

Phenolic substances represent crucial plant constituents possessing redox capacities, which can efficiently demonstrate antioxidant functions, including eliminating and suppressing reactive oxygen species (ROS), plus metal chelation [56]. It has been well documented in previous studies that Fu brick tea is rich in polyphenols. Gallic acid, catechin and epicatechin are the characteristic polyphenols and catechins of FBTE. The abundant polyphenols and catechins serve as the primary material basis for the antioxidant activity of pure FBTE [57,58]. As reported by Chen et al., the total phenolic content, total flavonoid content, ABTS radical scavenging activity, and DPPH radical scavenging activity of Fu brick tea produced in Xi’an were determined to be 34.85 mg GAE/g d.w., 37.24 mg RE/g d.w., 48.52 mg VCE/g d.w., and 90.21 mg VCE/g d.w., respectively [57].

As illustrated in Figure 3C, the variations in total phenolic quantity (TPC) for Z/CA/FBTE mixed films containing diverse FBTE ratios are displayed. As the FBTE proportion rises, TPC likewise steadily grows. Once the FBTE mass fraction reaches 0 wt%, the TPC of these films remains at a fairly minimal state. As the FBTE mass fraction reaches 8 wt%, TPC climbs by 72.96%. This occurs because the Fu brick tea extract contains numerous phenolic molecules like tea polyphenols and caffeine, which consequently boosts the total phenolic levels of the blended film [57]. The antioxidant potential of these composite films was assessed via DPPH and ABTS radical quenching tests (as shown in Figure 3D). The Z/CA/FBTE-0% sample demonstrated the minimal levels of DPPH and ABTS radical inhibition. As the FBTE content rose, these scavenging efficiencies gradually improved. Significantly, when the extract proportion attained 8 wt%, the DPPH and ABTS radical inhibition rates increased by 82.62% and 79.32%, respectively. Such improvement might be attributed to the occurrence of phenolic substances in FBTE, such as organic acids, gallic acid, catechin, and epicatechin. The phenolic hydroxyl moieties within these molecules can release electrons, thus efficiently suppressing free radicals [59]. Comparable findings were reported in a research concerning the creation of a preservation film using chitosan and guar gum as the primary components, with watermelon rind extract acting as both a cross-linking reagent and an active additive. When the watermelon rind extract ratio reached 4 wt%, the hybrid film’s DPPH radical quenching rate reached 83.24%. Watermelon rind extract is rich in bioactive polyphenols, which can capture free radicals and terminate the free radical chain reaction [20].

### 3.8. Analysis of the Water Contact Angle (WCA)

The water contact angle serves as a primary wetting characteristic metric for assessing the hydrophilic or hydrophobic nature of packaging substrates. Typically, a reduced WCA suggests that the film surface exhibits increased hydrophilic behavior [43]. As illustrated in Figure 3E, as the FBTE concentration rises, the water contact angle steadily rises, demonstrating an improvement in the hydrophobic attributes of the membrane. Upon reaching the highest FBTE level (8 wt%), the water contact angle surpasses that of the Z/CA/FBTE-0% sample by 49.27%. This phenomenon likely arises because incorporating FBTE causes the film surface to become rougher and more irregular. Such variations in surface topography might account for the observed alterations in contact angles among different films [60]. Within the research concerning konjac glucomannan/zein active films containing tea polyphenol–iron nanoparticles for strawberry conservation [43], it was noted that incorporating tea polyphenol–iron nanoparticles (TP-Fe NPs) further boosts hydrophobicity, primarily because of the elevated concentration of tea polyphenols within the matrix.

### 3.9. Antibacterial Activity

The proliferation of bacteria often results in food deterioration and foodborne illnesses, posing a significant challenge in food preservation. Incorporating antimicrobial agents into packaging layers is a strategy to inhibit microbial development [61]. As illustrated in Table 2, images of antibacterial efficacy are displayed, depicting the radius of the inhibition circle and the impact of various FBTE concentrations on *Staphylococcus aureus* (*S. aureus*) plus *Escherichia coli* (*E. coli*). With increasing FBTE loading, the measured inhibition zone diameters show a gradual increasing trend, indicating a mild enhancement in antibacterial response. The inhibition circle radius for Z/CA/FBTE-0 wt% is not distinct, suggesting that the fundamental composite membrane (lacking FBTE) possesses low antibacterial activity. When the FBTE proportion reaches 8 wt%, the inhibition circle radii for *S. aureus* and *E. coli* grow by 61.59% and 62.57%, respectively. This is due to bioactive elements like tea polyphenols and caffeine abundant within the Fu brick tea extract. These bioactive compounds impair bacterial membrane integrity and inhibit the respiratory metabolism of bacterial cells, collectively conferring potent antibacterial activity [23]. In the research regarding the antimicrobial and antioxidant impacts of potato peel extract in active edible films, it was observed that the hybrid film with potato peel inclusion had a clear inhibitory influence on *S. aureus* and *E. coli* [62]. Likewise, in the investigation of smart polycaprolactone/gelatin/zein nanofiber films loaded with thymol/alizarin, He et al. [63] noted that with the rise in thymol concentration, the inhibition circle radius steadily grows, and the antimicrobial efficiency is boosted.

### 3.10. Soil Degradation Test of the Composite Film

Soil biodegradation is a complex process affected by various factors, including the chemical properties of components, environmental conditions and microbial metabolic activities. Previous studies have demonstrated that biodegradable materials can help mitigate ecological pollution caused by synthetic packaging waste [64]. As presented in Table 3, morphological changes in composite films with different FBTE contents were continuously monitored and recorded throughout the 28-day soil burial test. The films exhibited progressive morphological alterations under soil conditions: in the initial stage (Day 1–7), changes occurred in the surface microstructure and color of the films; in the middle stage (Day 7–21), visible fragmentation of the films became more obvious; in the late stage (Day 21–28), the size of film fragments was further reduced. Existing research [65] has reported that biodegradable composite films composed of polylactic acid (PLA), natural rubber (NR) and rice straw (RS), as well as PLA/NR blends filled with RS, undergo obvious morphological degradation during soil incubation.

### 3.11. Measurement of the Peroxide Value (POV) in Lard

The peroxide value (POV) represents the quantitative assessment of hydrogen peroxide generated during the early phases of lipid rancidity. A higher POV indicates elevated oxygen permeability [23]. Figure 3F illustrates the fluctuations in POV throughout 20-day storage for the exposed group, the aluminum foil bag group, and the FBTE film group. Regarding the exposed group, POV rose markedly over time, peaking at the 20th day. This suggests that under ambient conditions, the rate of oil degradation accelerates swiftly. The POV level in the aluminum foil bag group stayed relatively minimal during the duration, with a stable variation, suggesting that aluminum foil can efficiently obstruct oxygen and various elements to suppress lipid oxidation. Contrary to the Z/CA/FBTE composite film group, as the FBTE ratio rose from 0 wt% to 6 wt%, the POV typically exhibited a decreasing pattern simultaneously, suggesting that the Z/CA/FBTE-6% film possessed the optimal oxidation-inhibiting performance. Nevertheless, when the extract ratio was raised further, the POV for the Z/CA/FBTE-8% film surged considerably. The primary cause for this discrepancy stems from the antioxidant molecules in FBTE capturing free radicals and postponing the lipid oxidation procedure [66]. Likewise, within the research concerning the synthesis and performance evaluation of composite films from corn starch, κ-carrageenan, and ethanol extract of onion peel [67], it was observed that when the mass fraction of EEOS reached 3%, the film’s antioxidant impact on fats was markedly enhanced.

## 4. Conclusions

This study developed bio-composite film utilizing Z and CA as base components, with FBTE loadings ranging from 0 wt% to 8 wt%. From a physical and chemical perspective, as FBTE concentration rose, considerable improvements were noted in film thickness, water contact angle (WCA), and total phenolic content (TPC). Such findings highlight the efficiency of FBTE in altering the structural and chemical properties of these hybrid films. Additionally, concerning antioxidant performance, FBTE notably enhanced the radical scavenging capacity of the films, and the 8 wt% FBTE sample achieved the highest antioxidant activity among all formulations. Nevertheless, the excessive incorporation of FBTE at 8 wt% led to deteriorated mechanical properties, increased WVP, and more porous, less uniform microstructure observed via SEM. SEM photographs clearly demonstrate that with elevated FBTE amounts, the surface and cross-sectional morphologies of the films become increasingly porous. Regarding antibacterial efficiency, FBTE exhibited a stronger inhibitory effect on Gram-negative bacteria (*E. coli*) compared to Gram-positive bacteria (*S. aureus*). Furthermore, lipid peroxidation assays indicated that the composite membrane with 6 wt% FBTE effectively suppressed the rancidity of lard. Overall, the 6 wt% FBTE formulation achieved the optimal comprehensive balance of antioxidant activity, mechanical performance, barrier properties and microstructure, and exerted the most prominent protective effect against lard oxidation. Observation during soil burial degradation showed that the fragments of composite films gradually reduced in size over time. In summary, Z/CA/FBTE composite films possess antioxidant and antibacterial properties and can retard lipid oxidation, showing potential for application in active packaging of solid lipid-based foods.

## Figures and Tables

**Table 1:** Thickness, chromatic parameters, transparency, and images of blended films at various FBTE concentrations.

Concentration of the Extract	Thickness	L*	a*	b*	Opacity/%	Picture
0%	0.105 ± 0.02 ^e^	76.14 ± 0.24 ^a^	4.17 ± 0.19 ^d^	65.89 ± 0.33 ^a^	4.59 ± 0.02 ^e^	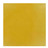
2%	0.108 ± 0.47 ^d^	74.82 ± 0.18 ^b^	5.96 ± 0.54 ^c^	62.96 ± 0.44 ^e^	5.22 ± 0.16 ^d^	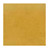
4%	0.1170 ± 0.67 ^c^	71.27 ± 0.24 ^b^	8.34 ± 0.27 ^c^	60.23 ± 0.09 ^d^	6.15 ± 0.46 ^c^	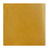
6%	0.121 ± 0.82 ^b^	69.48 ± 0.24 ^c^	9.81 ± 0.76 ^b^	58.80 ± 0.67 ^c^	7.01 ± 0.23 ^b^	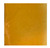
8%	0.146 ± 0.11 ^a^	61.73 ± 0.62 ^d^	15.46 ± 0.07 ^a^	55.46 ± 0.25 ^b^	8.22 ± 0.32 ^a^	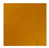

Values for a–e are presented as means ± SD. Distinct letters within the same row signify a significant difference (*p* < 0.05).

**Table 2:** Antibacterial efficacy of these integrated films regarding *S. aureus* and *E. coli*.

Composite Film	*S. aureus* (mm)		*E. coli* (mm)	
**Z/CA/FBTE-0%**	1.26 ± 0.12 ^d^	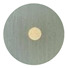	1.28 ± 0.04 ^e^	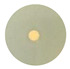
**Z/CA/FBTE-2%**	1.27 ± 0.57 ^d^	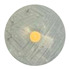	1.49 ± 0.77 ^d^	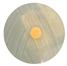
**Z/CA/FBTE-4%**	1.92 ± 0.73 ^c^	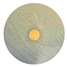	2.10 ± 0.59 ^c^	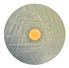
**Z/CA/FBTE-6%**	2.79 ± 0.18 ^b^	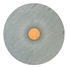	2.82 ± 0.64 ^b^	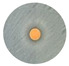
**Z/CA/FBTE-8%**	3.28 ± 0.17 ^a^	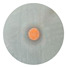	3.42 ± 0.62 ^a^	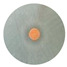

Mean ± SD are utilized to represent a–e data. Distinct letters within the same row signify a notable disparity (*p* < 0.05).

**Table 3:** Morphological transformations of Z/CA/FBTE films following 28-day deterioration.

Degradation Time	1 Day	7 Day	14 Day	21 Day	28 Day
**The change in the appearance of the film**	** 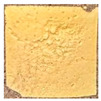 **	** 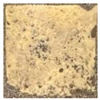 **	** 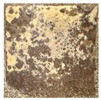 **	** 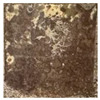 **	** 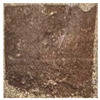 **

## Data Availability

The original contributions presented in the study are included in the article. Further inquiries can be directed to the corresponding author.
